# Targeting the Hedgehog Pathway in Cancer: Current Evidence and Future Perspectives

**DOI:** 10.3390/cells8020153

**Published:** 2019-02-12

**Authors:** Daniel Girardi, Adriana Barrichello, Gustavo Fernandes, Allan Pereira

**Affiliations:** Division of Medical Oncology, Hospital Sírio-Libanês, Brasilia 70200-730, Brazil; dribarrichello@hotmail.com (A.B.); gustavo.hemato@gmail.com (G.F.); allan.pereira@hsl.org.br (A.P.)

**Keywords:** Hedgehog, targeted therapy, SMO inhibitor, GLI

## Abstract

The Hedgehog pathway (HhP) plays an important role in normal embryonic development and its abnormal function has been linked to a variety of neoplasms. Recently, the complex mechanisms involved in this pathway have been deciphered and the cross talks with other important pathways involved in carcinogenesis have been characterized. This knowledge has led to the development of targeted therapies against key components of HhP, which culminated in the approval of vismodegib for the treatment of advanced basal cell carcinoma in 2012. Since then, other compounds have been developed and evaluated in preclinical and clinical studies with interesting results. Today, several medications against components of the HhP have demonstrated clinical activity as monotherapies and in combination with cytotoxic treatment or other targeted therapies against mitogenic pathways that are linked to the HhP. This review aims to clarify the mechanism of the HhP and the complex crosstalk with others pathways involved in carcinogenesis and to discuss both the evidence associated with the growing number of medications and combined therapies addressing this pathway and future perspectives.

## 1. Introduction

The Hedgehog pathway (HhP) plays a fundamental role in embryonic development, tissue patterning, and wound healing [1]. Aberrant functioning of this pathway is related to several congenital abnormalities and the development of cancer in several organs [2,3].

The idea that alterations in the HhP are linked to carcinogenicity came from the discovery of activating mutations in this pathway in patients with basal cell carcinoma (BCC), medulloblastoma and rhabdomyosarcoma [4,5,6,7]. These findings led to a better understanding of the pathway and the development of targeted therapies directed against their effectors [8,9].

In this review, we focus on the characterization of the HhP, its relation with other pathways related to the development of cancer and especially on treatment strategies as a way to fight cancer.

## 2. Characterization of the HhP and Its Relation with Carcinogenesis

**Figure 1:**
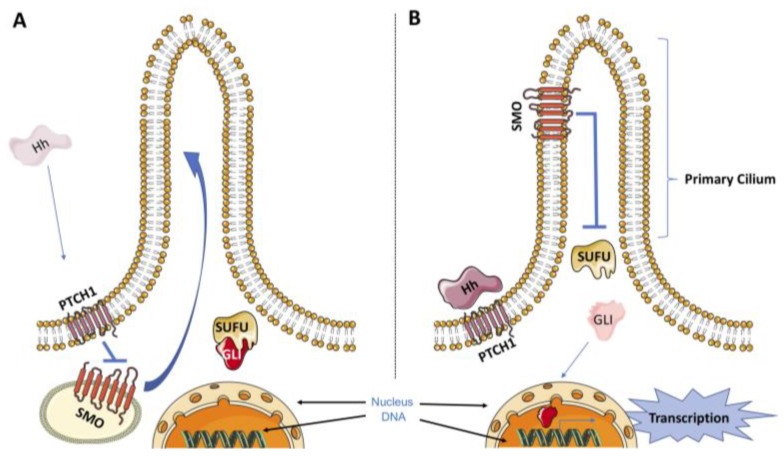
A simplified model for canonical HhP. (**A**) The Hedgehog receptor PTCH1 inhibits SMO signaling in the absence of the Hedgehog ligand, which turns off the Hedgehog signaling pathway. (**B**) In the presence of Hedgehog ligand, PTCH1 stops inhibiting SMO, resulting in the translocation of SMO to the primary cilium. Once activated in the primary cilium, SMO promotes the release of GLI from the SUFU, which allows GLI to enter the nucleus to initiate the transcription program related to HhP. Dysregulation of the HhP has been associated with carcinogenesis.

The HhP is very complex and can be divided into two different pathways: canonical and noncanonical. The canonical HhP is initiated by the release of three ligands named Sonic Hedgehog (SHH), Desert Hedgehog (DHH), and Indian Hedgehog (IHH) [10]. In the absence of these ligands, the 12-pass transmembrane receptor Patched1 (PTCH1) exerts an inhibitory effect on the transmembrane transducer smoothened (SMO) [11]. Binding of Hedgehog ligands to PTCH1 relieves the repression of SMO by PTCH1, which results in the translocation of SMO to the primary cilium. The primary cilium is a membrane-encased protrusion usually described as a single nonmotile cilium present in a variety of vertebrate cells. In humans, for instance, virtually all other cells have a primary cilium, with the exception of sperm, epithelia cells in the bronchi and oviducts, and ependymal cells that line the brain vesicles. The translocation of SMO to the primary cilium, in turn, initiates an intracellular signal cascade that promotes the activation of glioma-associated oncogene (GLI) transcription factors [12]. There are three members of the GLI transcription factor family (GLI1, GLI2 and GLI3) that share a similar DNA-binding domain [13]. Once activated in the primary cilium, GLIs dissociate from the suppressor of fused (SUFU), which is a key cytoplasmic negative regulator of the HhP, and translocate into the nucleus to initiate the transcription program related to the HhP (Figure 1) [14]. In the absence of HH ligands, PTCH1 exerts a repressive effect on SMO by preventing its accumulation in the primary cilium. In this state, SMO is not capable of activating GLI transcription factors, which are bound to SUFU and retained in the cytoplasm. In the cytoplasm, the degradation of GLI proteins by the proteasome can occur through phosphorylation by protein kinase A (PKA), casein kinase 1 (CK1) and glycogen synthase kinase 3β (GSK3β) [15,16].

In the noncanonical HhP, activation of the GLI transcription factors can occur independently of the upstream components of the HhP by cross talk with other signaling cascades [16]. Multiple cross talk and synergistic interactions between HhP components and other important oncogenic pathways have been shown to activate HhP and have been implicated in several types of cancer. For example, cross talk between HhP and the mechanistic target of rapamycin (mTOR) pathway has been described in esophageal carcinoma in which GLI1 appears to be activated by ribosomal protein S6 kinase 1 (S6K1) through direct phosphorylation at SEr84 [17]. Conversely, another study demonstrated that V-akt murine thymoma viral oncogene homolog 1 (AKT1) is a direct transcriptional target of GLI1 [18].

Cytotoxic agents, such as radiotherapy and cytokines, can also activate and upregulate the expression of GLI1 independent of canonical pathway activation. For example, tumor necrosis factor alpha (TNF-α) and interleukin-1β (IL-1β) can upregulate the expression of GLI1 through the nuclear factor kappa light chain enhancer of activated B cells (NF-κB) pathway in pancreatic and breast cancer cells [19,20]. Interestingly, evidence now shows that NF-κB subunit p65 can bind directly to the GLI1 promoter region, and inhibition of NF-κB decreased GLI1 activity in breast cancer cells [20]. Transforming growth factor-beta (TGF-β) has also been demonstrated to upregulate GLI1 and GLI2 via the SMAD3 pathway and may also lead to GLI protein accumulation in cancer cells [21,22]. In pancreatic cancer cell lines resistant to Hedgehog inhibitors, the pharmacologic blockade of TGF-β has been shown to inhibit cell proliferation [21]. Similarly, inhibition of TGF-β also contributed to reduced tumor volume in preclinical models of SMO-induced BCC and reduced tumor cell invasion in gastric cancer cell models [22,23].

The RAS/RAF/MEK/ERK pathway can also upregulate and activate GLI transcriptional activity. An interesting study demonstrated that oncogenic KRAS was able to increase GLI activity in pancreatic adenocarcinoma cell lines, and this effect was inhibited by MEK inhibitors [24]. Another study demonstrated that EGFR synergizes with GLI in the carcinogenesis process via the RAS/RAF/MEK/ERK axis and that in vitro dual inhibition of epidermal growth factor receptor (EGFR) and GLI led to a more potent reduction in cell proliferation in BCC cells than either inhibitor alone [25]. Finally, a study shows that c-MYC can directly regulate GLI1 by interacting with the 5′-regulatory region of GLI1 and that inhibition of c-MYC can decrease GLI1 mRNA [26]. This study also provides evidence that the use of GLI1 inhibitors was able to increase tumor control of Burkitt lymphoma cells [26].

## 3. Other Implications of the HhP and Cancer

As mentioned above, upregulation and overexpression of the HhP are related to several cancer types, such as lung, head and neck, esophagus, colon, pancreas, glioma, breast, ovarian and cervical [1]. Recently, some clinical implications of this upregulation and overexpression have been elucidated. For example, GLI1 activation was associated with distant metastasis and poor outcomes in patients with head and neck squamous cell carcinoma (HNSSC) [27]. Similar findings were described in esophageal squamous cell carcinoma in which GLI1 overexpression was associated with node metastasis and poor prognosis [28]. Upregulation of GLI1 expression in colon cancer was correlated with node metastasis, T-stage and postoperative live metastasis-free survival periods [29]. GLI2 expression was also correlated with poor outcomes in acute myeloid leukemia. A patient cohort indicated that patients with GLI2 overexpression had significantly worse outcomes in terms of event-free survival (EFS), relapse-free survival (RFS), and overall survival (OS) [30].

The HhP is also implicated in resistance to cancer therapies. For example, a study demonstrated the role of the HhP in chemoresistant ovarian cancer cell lines, and the use of SMO antagonists was able to sensitize chemotherapy-resistant cell lines to paclitaxel [31]. One of the reasons why tumors can become resistant to treatment is the epithelial-to-mesenchymal transition (EMT), which has been associated with the HhP. Multiple genes and proteins have been implicated in this mechanism, including those of the HhP, the NOTCH and WNT pathways [32,33] and the transcription factors ZEB1, ZEB2, SNAI1, SLUG and TWIST1 [34,35,36]. EMT is a process that occurs both in normal cells (during wound repairs and embryonic development) and in cancer cells, where it is implicated in resistance to chemotherapy and radiotherapy and increased migratory and invasive properties [34,37,38,39,40]. During the EMT process, cancer cells appear to acquire a stem cell-like phenotype that portents the capacity of self-renewal, resistance to cancer therapies and repopulation after a cytotoxic treatment [41,42,43,44]. The GLI transcriptional factors mediate the stem cell-like phenotype by regulating the transcription of genes involved in this signature, such as NANOG, octamer binding transcription factor 4 (OCT4), SOX2, *BMI1,* WNT-2, and Kruppel-like factor 4 (KLF4) [45,46].

Preclinical data have shown that in HNSSC cells, the expression of GLI transcription factors is increased in the population of cells that were resistant to EGFR inhibitors and radiotherapy [47,48]. These cell lines expressed higher levels of HhP genes and a stem cell-like phenotype [1]. This process was also described in other cancer types, such as lung, esophagus, gastric and colorectal cancers, in which transcriptional activation of genes related to EMT and stem cell-like phenotype were mediated by the HhP through GLI [49,50,51,52]. In a lung cancer model, HhP inhibition was able to reverse EGFR resistance and the stem cell-like phenotype [49].

## 4. SMO Inhibitors

A great deal of effort has been focused on targeting SMO in particular [53]. To date, two SMO inhibitors (sonidegib and vismodegib) have received US Food and Drug Administration (FDA) approval for treating BCC, while many clinical trials are being conducted to evaluate the efficacy of this exciting class of targeted therapies in a variety of cancers. Table 1 summarizes the clinical trials that evaluated SMO inhibitors against a variety of cancer types.

### 4.1. Cyclopamine

Cyclopamine (11-deoxojervine), a name derived from the word cyclopia, is a naturally occurring chemical isolated from the corn lily (*Veratrum californicum*) and belongs to the family of steroidal alkaloids.

In the 1950s, a group of ranchers in Idaho were surprised when their sheep gave birth to lambs with only one eye. After decades of research, they discovered that the cause of the deformity was the corn lily ingested by the pregnant sheep. Four decades later, the relationship of the SHH gene with cyclopamine was discovered. Upon experimentation, they recreated cyclopia by silencing the SHH gene and then connected their results to the cycloptic sheep noted four decades earlier.

In 1998, the first studies showing that cyclopamine inhibits SHH signal transduction were published [91,92], but the potential effects of cyclopamine or synthetic derivatives on cancer were reported in 2000 [93].

Cyclopamine has a high affinity for SMO, and upon binding, it inhibits the signal. It was the first compound found to inhibit HhP signaling and has been very valuable for understanding the function of Hedgehog signaling. Cyclopamine is widely used as a Hedgehog inhibitor in cell and murine models of various tumors [94,95,96,97]. However, the poor solubility and the low potency of cyclopamine prevent its clinical usage. A cyclopamine synthetic analog with greater solubility, cyclopamine tartrate (CycT), was reported for its activities in HhP signaling-mediated cancer in vitro and in vivo (mice) but had uncertain results and is still not used in clinical practice [98,99].

### 4.2. Vismodegib (GDC-0449)

Vismodegib is the first FDA-approved SMO inhibitor for the treatment of advanced and metastatic BCC. Currently, vismodegib and many other SMO inhibitors are being investigated in clinical trials in a range of advanced cancers [73,100]. This medication binds to and directly inhibits SMO, blocking the signal transduction in the HhP.

The international, open-label STEVIE trial is the largest study of safety and efficacy of vismodegib in BCC [55]. This trial allocated 1215 patients with locally advanced and metastatic BCC from 36 countries. The response rates were 68.5% in patients with locally advanced BCC and 36.9% in patients with metastatic BCC, but serious side effects occurred in 289 patients (23.8%), with death in 46 patients (3.8%).

It is not clear whether treatment with vismodegib increases the risk of cutaneous squamous cell carcinoma. A small case control study suggested this association and showed that in the vismodegib group, the risk of new cases of squamous cell carcinoma increased eightfold [101]. Controversially, another case control study did not demonstrate this association [102]. Even with these controversial data, continuous skin surveillance should be performed after the initiation of this therapy.

Acquired resistance was identified in 21% of the patients treated while undergoing continuous vismodegib treatment, with a mean time to detected regrowth by clinical examination of 56.4 weeks [103].

### 4.3. Sonidegib (LDE-225)

Sonidegib is a direct inhibitor of SMO, blocking the signal transduction in the HhP. In the phase I study, sonidegib had an acceptable safety profile in patients with advanced solid tumors and exhibited antitumor activity in advanced BCC and relapsed medulloblastoma [104]. In a phase 2 study, it was shown that 800 mg daily is not more efficacious than 200 mg and causes more adverse effects (grade 3/4 effects adverse of 43.0% vs. 64.0%). As BCC is a slow growing tumor, even after 30 months, the median OS had not been reached. The estimated 2-year OS rates in patients taking 200 mg were 93.2% for advanced disease and 69.3% for those with metastases, and few patients had a complete response [74].

In the comparison of sonidegib with vismodegib, a recent meta-analysis has shown that in locally advanced BCC, overall response rates (ORRs) were similar for vismodegib and sonidegib (69% vs. 57%), but complete response rates were not (31% vs. 3%). In metastatic disease, the ORR of vismodegib was 2.7-fold higher than the ORR of sonidegib (39% vs. 15%). Side effects were similar in both groups, except for upper gastrointestinal symptoms, which were higher in patients receiving sonidegib [105].

Regarding the tumors resistant to Hedgehog inhibitors, a study showed that patients with advanced BCCs who were previously resistant to treatment with vismodegib similarly demonstrated the same treatment resistance with sonidegib [106].

Currently, sonidegib is under active clinical phase I/II investigation in both solid and hematologic malignancies. Studies that have examined sonidegib alone or in combination with targeted therapy have demonstrated a synergistic mediated effect [107,108,109].

### 4.4. Saridegib (IPI-926)

Saridegib is a potent and specific inhibitor of SMO derived from cyclopamine. In vitro and in vivo studies have shown the activity of this drug in many types of cancer, such as ovarian, medulloblastoma and head and neck [1]. Furthermore, evidence from a genetically engineered mouse model of pancreatic cancer demonstrated that saridegib can deplete tumor-associated stromal tissue and increase intratumoral mean vessel density [110]. These changes resulted in enhanced delivery of concurrently administered systemic chemotherapy, leading to a decreased tumor burden and prolonged survival in this mouse model.

A multicenter phase Ib study evaluated saridegib in combination with FOLFIRINOX in patients with advanced pancreatic cancer [79]. The objective response rate was high (67%), and patients receiving saridegib maintenance showed further declines in CA19-9 levels even after FOLFIRINOX discontinuation. However, the study closed early when a separate phase II trial of saridegib plus gemcitabine indicated detrimental effects of this combination.

### 4.5. Taladegib (LY2940680)

Taladegib is an antagonist of the Hedgehog ligand cell surface receptor SMO with potential antineoplastic activity. Recently, a study demonstrated the in vivo efficacy of taladegib in a mouse medulloblastoma allograft model [111].

Taladegib has been studied in several trials involving solid tumors, such as colon cancer, breast cancer and rhabdomyosarcoma. However, a phase 1 trial suggests that taladegib is effective only in BCC. This trial evaluated several advanced solid tumors, and collectively, the clinical responses in BCC patients had an estimated ORR of 46.8%. This study also suggested potential benefit of taladegib not only in Hedgehog treatment-naïve patients but also in patients who were previously treated with Hedgehog inhibitor therapy [87].

### 4.6. Glasdegib (PF-04449913)

Glasdegib is an oral, potent, selective inhibitor of the HhP that functions through binding to the SMO receptor [112]. This molecule prevents the translocation of SMO into primary cilia and prevents SMO-mediated activation of downstream Hedgehog targets [113]. In preclinical studies, glasdegib inhibited SMO in vitro and induced significant antitumor activity in vivo.

Its use seems to be attractive in myeloid malignancies since it has demonstrated that glasdegib inhibition of SMO reduces the expression of key intracellular leukemia stem cell regulators [114]. In addition, the administration of glasdegib also resulted in a significant reduction in leukemic stem cell (LSC) burden in xenograft models, inhibition of HhP signaling, and a reduction in cell populations expressing LSC markers [115]. A phase 1 study suggested that glasdegib had no effect on solid tumors. In this study, eight patients (34.8%) achieved stable disease, and none had a complete or partial response. Three patients with disease progression at enrollment had prolonged disease stabilization (≥6 months) [90].

### 4.7. Itraconazole

Itraconazole, an FDA-approved antifungal drug, appears to act on the essential HhP component SMO by a mechanism distinct from that of cyclopamine and other known SMO antagonists [116].

In clinical trials, itraconazole was tested in an open-label, phase II trial for the treatment of BCC in 29 patients. Itraconazole reduced cell proliferation by 45%, HhP activity by 65%, and tumor area by 24% [84]. In another randomized phase II clinical trial of metastatic castration-resistant prostate cancer, 46 chemotherapy-naïve patients were enrolled, of whom 29 received high-dose itraconazole treatment (600 mg/day), and 17 received low-dose (200 mg/day) itraconazole treatment. Prostate-specific antigen progression-free survival (PFS) rates at 24 weeks were 48% and 11.8% with median PFS of 11.9 and 35.9 weeks in the high- and low-dose arms, respectively [86].

Additional retrospective studies supported the survival advantage of itraconazole treatment in refractory malignancies, including ovarian clear cell, triple-negative breast, pancreatic and biliary tract cancers, compared with previous reports [117,118,119,120].

## 5. GLI Inhibitors

As demonstrated above, the HhP can be activated downstream of SMO, and multiple cross talk between the HhP and other mitogenic pathways converges to amplify and activate GLI transcriptional factors. This cross talk is also one of the causes of resistance to SMO inhibitors, along with mutations in SMO [121,122,123]. Strategies to address this mechanism and overcome resistance to SMO inhibitors are targeted therapies against GLI and combination with targeted agents against other oncogenic pathways involved in GLI activation [15].

GANT58 and GANT61 are GLI antagonists that can interfere with GLI translocation into the nucleus and can prevent DNA binding [124]. GANT61 is the most studied and efficient antagonist that can bind to the zinc finger regions 2 and 3 of GLI1 and GLI2 [1]. Several preclinical studies demonstrated antitumor activity in many cancer types, such as lung, acute myeloid leukemia, rhabdomyosarcoma, neuroblastoma, breast cancer, colon, prostate, melanoma, and pancreatic cancer [15,124,125,126,127,128,129]. The main effects of GANT61 are the interference with the cell cycle by induction of G1 arrest and expression of p21 [127,130,131]; the cytotoxic activity through activation of Fas signaling and decreased levels of Bcl2 (an antiapoptotic protein) [15,125,132,133]; the attenuation of the EMT process slowing down cell migration [131,132,134,135]; the decrease in the transcription of genes related to stem cell phenotype, such as NANOG, SOX2, OCT4 and c-MYC [15,136]; and the increase in the production of inflammatory cytokines such as IL8 and MCP1, which increases monocyte recruitment [137].

Arsenic trioxide (ATO) is known for its use in acute promyelocytic leukemia; however, it is also a GLI inhibitor [138]. The mechanism of action is the inhibition of GLI2 by blocking the trafficking in and out the primary cilium, which is necessary for GLI2 activation [139]. In preclinical studies, ATO was able to reduce the growth of medulloblastoma allografts [139]. A similar mechanism of action was described for pirfenidone, which is a drug approved for the treatment of idiopathic pulmonary fibrosis [140]. This drug is also capable of destabilizing GLI2 and decreasing the expression of TGF-β [141]. In fact, some recent preclinical studies have shown promising antitumor results in several cancer types, such as lung, breast, pancreas, glioma and hepatocellular carcinoma [142,143,144,145,146,147].

Four Hedgehog pathway inhibitors (HPIs) with unique mechanisms of action downstream of SMO that are capable of modulating GLI activity have been recently described [15]. HPI-1 likely increases GLI repression through PKA phosphorylation, HPI-2 and HPI-3 modulate the activity of GLI2, and HPI-4 interferes with ciliogenesis and therefore with GLI activation [148]. Pyrvinium, an antihelminthic, is another drug that has been shown to have inhibitory effects on the HhP. The proposed mechanism of action is the activation of CK1, which facilitates the phosphorylation of GLI proteins and consequent degradation [149]. Imiquimod, an agonist of toll-like receptor (TLR) 7 and TLR8, can also modulate GLI activity by activating PKA and subsequent degradation of GLI proteins [150]. Nanoquinacrine, a spherical nanoparticle form of quinacrine, also interferes with the HhP by increasing the expression of GSK3β and interfering with the binding of GLI1 and DNA [151]. Recently, the bromodomain and extra terminal (BET) proteins, specifically the bromodomain-containing protein 4 (BRD4), have been described and associated with HhP inhibition. These molecules can bind directly to GLI promoter regions on DNA and epigenetically regulate its transcription. Two preclinical studies have demonstrated the antitumor properties of these protein inhibitors [152,153].

## 6. Combination Therapy

The characterization of the noncanonical pathway elucidated cross talk between the HhP and other oncogenic pathways, such as mTOR, EGFR, MAPK, NF-κB and TGF-β. Consequently, several studies evaluated the combination therapy between agents targeting HhP components and agents addressing components of these other pathways. A promising combination is dual inhibition of the HhP and mTOR pathways. Two preclinical studies have demonstrated the efficacy of GANT61 and mTOR inhibitor combination in myeloid leukemia cells and in rhabdomyosarcoma cells [127,154]. In vitro studies have also demonstrated that blockage of mTOR pathways improves the effect of Hedgehog inhibitors in esophageal and head and neck cancers [17,47]. The combination of an SMO inhibitor (sonidegib) with a PI3K inhibitor (NVP-BKM120 or NVP-BEZ235) delayed the development of resistance to SMO inhibitor in medulloblastoma animal models [123]. Similar findings have been described in pancreatic cancer, demonstrating the in vitro benefit of combining mTOR and SMO inhibitors [36], and in biliary tract cancer cells [155]. Another combination that shows promising results in preclinical studies involves EGFR and the HhP. As mentioned above, dual inhibition of EGFR and GLI showed efficient antitumor activity in BCC cell lines of mice with an activated HhP [25].

The combination of targeted therapy against the HhP and cytotoxic agents has also demonstrated promising results in preclinical trials. GANT61 was able to increase the radiosensitivity of renal cell carcinoma cells, and this was also noted in the combination therapy involving GANT61 and hypoxia-inducible factor 2α (HIF2α) inhibitor [15,156]. Similar findings were observed in the HNSCC model, in which an HhP blockade with cyclopamine in chronically irradiated or EGFR-resistant cell lines increased sensitivity to radiotherapy [47] and in non-small cell lung cancer preclinical models, in which vismodegib increase in vivo radiation efficacy [157].

Preclinical evidence that Hedgehog inhibition may sensitize acute myeloid leukemia cells to cytarabine or azacytidine provided rationale for evaluating glasdegib in combination with chemotherapeutic agents. A recent phase 2 trial evaluated the combination of glasdegib with cytarabine and daunorubicin in patients with acute myeloid leukemia or high-risk myelodysplastic syndromes. This trial demonstrated that the combination is feasible and safe with clinical activity. An ongoing phase 3 trial will help clarify the activity of this combination [89]. Itraconazole associated with pemetrexed has been studied in patients with recurrent non-small cell lung cancer. A phase 2 trial was designed to evaluate this combination. The study was stopped early because of the increased use of pemetrexed in the first-line setting, but preliminary results of the 23 enrolled patients had already shown benefit with this combination. At 3 months, 67% of the patients on itraconazole plus pemetrexed were progression-free versus 29% on the control arm of pemetrexed alone. The median PFS was 5.5 months (itraconazole) versus 2.8 months (control). OS was longer in patients receiving itraconazole (median 32 months) versus control (8 months) [85].

However, in some clinical trials, the results have been disappointing. Two phase II trials failed to show the benefit of the combination of HhP inhibitor and chemotherapy. One study failed to show improvement in PFS with vismodegib in combination with standard treatment (FOLFOX or FOLFIRI and bevacizumab) versus standard treatment alone (hazard ratio (HR): 125, p=0.28) in patients with previously untreated metastatic colorectal cancer [70]. The other study evaluated vismodegib as a maintenance treatment in patients with ovarian cancer after second or third complete remission. The study fails to show a significant difference in PFS between vismodegib and placebo (median PFS 7.5 versus 5.8 months, respectively, HR 0.79; 95% confidence interval (CI), 0.46-1.35) [72]. A phase 1b trial evaluated the combination of cetuximab and saridegib for HNSCC patients. One patient experienced a partial response, three stable disease and four disease progression [81]. A pilot trial evaluated the combination of ATO and itraconazole in five patients with metastatic BCC who experienced relapse after SMO inhibitor treatment. This combination was able to reduce GLI1 messenger RNA levels, but the best clinical response was stable disease [158].

## 7. Conclusion and Future Perspectives

The HhP is essential for embryogenesis and several other physiologic processes, such as wound healing. Recently, this pathway was also related to carcinogenesis in several cancer types. Targeted therapy against the HhP has shown impressive results for BCC, meduloblastoma and rhabdomyosarcoma but disappointing results in other histologies. In addition, resistance to SMO inhibitors in BCC is a reality that needs to be better comprehended. Recently, the elucidation and better understanding of this pathway have led to important discoveries. Multiple cross talk with other important oncogenic pathways opened new avenues to explore targeted therapy and overcome resistance. Several preclinical and animal models have revealed the efficacy and antitumor activity of targeted therapy against HhP components or combination therapy with other targeted agents, which have become promising in the fight against cancer.

Future clinical trials have the potential to add more knowledge to this scenario. Table 2 summarizes the clinical trials that are evaluating HhP inhibitors. This table comprehends single-agent treatments and combination treatments in many types of cancer. For example, a phase II trial is evaluating the combination of sonidegib (SMO inhibitor) with buparlisib (PI3K inhibitor) in patients with locally advanced or metastatic BCC (NCT02303041). Another interesting trial is evaluating the addition of vismodegib to neoadjuvant chemotherapy treatment in breast cancer patients (NCT02694224). Future research will certainly add more insight to the clinical role of this important pathway.

## Figures and Tables

**Table 1:** SMO inhibitors in malignant tumors tested in clinical trials completed by October 2018.

Study	Phase	Type of Cancer	Experimental Arm	Control Arm	Results of Primary EP
NCT02639117	Phase 1	Multiple BCC	Vismodegib + photodynamic therapy sessions + topical application of 20% 5-aminolevulinic acid (ALA)		Combination PDT-vismodegib therapy was overall well tolerated (50% dysgeusia, 50% myalgia, 75% flu-like symptoms) [54].
*STEVIE* NCT01367665	Phase 2	Locally advanced and metastatic BCC	Vismodegib		Serious side effects (grade ≥ 3) in 289 patients (23.8%) and death in 46 patients (3.8%) [55].
NCT01546519	Phase 1b	Advanced solid malignancies and hepatic impairment	Vismodegib		96.8% in all groups, experienced at least one AE. 67.7% of all AEs reported were grade 3 or 4 [56].
*ERIVANCE BCC* NCT00833417	Phase 2	Locally advanced and metastatic BCC	Vismodegib		ORR of 60.3% in patients with locally advanced BCC and 48.5% metastatic BCC [57].
*MIKIE* NCT01815840	Phase 2	Multiple BCC	A. Vismodegib 12 w - placebo 8 w - vismodegib 12 w B. Vismodegib 24 w - placebo 8 w - vismodegib 8 w		The mean number of BCC lesions at week 73 was reduced from baseline by 62.7% in group A and 54% in group B [58].
NCT00957229	Phase 2	Basal cell nevus syndrome (BCNS)	Vismodegib	Placebo	Reduced rate of new surgically eligible BCC (2 vs 34 per patient per year) [59].
NCT02115828	Phase 2	Metastatic castration-resistant prostate cancer	Vismodegib		Gli1 mRNA was significantly suppressed by vismodegib in both tumor tissue (57%) and benign skin biopsies (75%) [60].
NCT01631331	Phase 1	BCC	Neoadjuvant vismodegib		Reduction of the final surgical defect size by 34.8% compared with baseline [61].
*E1508* NCT00887159	Phase 2	Extensive stage small cell lung carcinoma	A. Cisplatin + etoposide B. Vismodegib C. Cixutumumab		The median PFS times in arms A, B, and C were 4.4, 4.4, and 4.6 months, respectively [62].
*VISMOLY* NCT01944943	Phase 2	Refractory or relapsed B-cell lymphoma or chronic lymphocytic leukemia	Vismodegib		The best overall response: DLBCL: 0 (0%), iNHL: 1 (16.7%), PCNSL: 0 (0%), CLL: (0%), all: 1 (3.2%) [63].
NCT01064622	Phase 1b/2	Metastatic pancreatic cancer	Gemcitabine + vismodegib	Gemcitabine plus Placebo	Median PFS was 4.0 and 2.5 months for GV and GP arms, respectively [64]
NCT01201915	Phase 2	BCC	Neoadjuvant vismodegib for 12 weeks for 12 weeks - 24 weeks of observation before excision for 8 weeks on - 4 weeks off - 8 weeks on		Complete histologic clearance was achieved by 42%, 16%, and 44% of patients in cohorts 1, 2, and 3, respectively [65].
NCT01195415	Phase 2	Metastatic pancreatic adenocarcinoma	Vismodegib plus gemcitabine		GLI1 and PTCH1 decreased in 95.6% and 82.6%, respectively [66].
NCT01267955	Phase 2	Advanced chondrosarcoma	Vismodegib		The 6-month clinical benefit rate was 25.6% [67].
NCT00822458	Phase 1	Medulloblastoma	Vismodegib		3 dose-limiting toxicities but no drug-related bone toxicity. The median vismodegib penetration in the CSF was 0.53 (ratio of the concentration of vismodegib in the CSF to that of the unbound drug in plasma) [68].
NCT00607724	Phase 1	BCC	Vismodegib		SUVmax decreased (median 33%, SD ± 45%) with metabolic activity normalizing or disappearing in 42% of lesions [69]
NCT00636610	Phase 2	Metastatic colorectal cancer	Vismodegib + FOLFOX or FOLFIRI + bevacizumab	Placebo + FOLFOX or FOLFIRI + bevacizumab	Median PFS hazard ratio (HR) was 1.25 [70].
NCT01209143	Phase 1b	Solid cancers	Vismodegib + rosiglitazone Vismodegib + oral contraceptive		Systemic exposure of rosiglitazone or oral contraceptive is not altered with concomitant vismodegib [71].
NCT00739661	Phase 2	Ovarian cancer	Vismodegib	Placebo	Median PFS in vismodegib and placebo groups were 7.5 months and 5.8 months, respectively [72].
NCT00607724	Phase 1	Solid tumor	Vismodegib		8 grade 3 adverse events in 6 patients. 1 patient withdrew from the study because of adverse events [73]
*BOLT* NCT01327053	Phase 2	BCC	Sonidegib 200 mg and 800 mg		Sonidegib 200 mg (approved dose), objective response rates were 56.1% (central) and 71.2% (investigator) in laBCC and 7.7% (central) and 23.1% (investigator) in mBCC [74].
NCT01125800	Phase 1/2	Medulloblastoma, rhabdomyosarcoma, neuroblastoma, hepatoblastoma, glioma, astrocytoma	Sonidegib 233 mg/m^2^ daily; 372 mg/m^2^ daily; 425 mg/m^2^ daily; 680 mg/m^2^ daily; 800 mg/m^2^ daily		The recommended phase II dose in pediatric patients was 680 mg/m^2^ once daily. The results were 4 complete responders (2 pediatric and 2 adult) and 1 partial response (adult) [75].
NCT01954355	Phase 1	Solid tumorovarian cancer	Sonidegib 400, 600 and 800 mg + paclitaxel		The recommended phase II dose was 800 mg in combination with paclitaxel [76].
NCT01208831	Phase 1	Advanced solid tumor cancers, medulloblastoma, BCC	Sonidegib		The recommended dose in East Asian patients (400 mg) was lower than in patients from Europe and the USA (800 mg and 250 mg, respectively, twice daily) [77].
NCT01579929	Phase 1	Lung cancer	Sonidegib + etoposide + cisplatin		Sonidegib 800 mg daily was the maximum tolerated dose when administered with EP [78].
NCT01383538	Phase 1	Advanced pancreatic adenocarcinoma	Saridegib + FOLFIRINOX		The combination was active and safe [79].
NCT01371617	Phase 2	Primary myelofibrosis	Saridegib		Nine out of fourteen patients (79%) did not respond. Saridegib is not active in myelofibrosis as monotherapy [80].
NCT01255800	Phase 1	Recurrent head and neck cancer	Saridegib + cetuximab		The recommended phase 2 dose was 160 mg, the same as the single-agent saridegib maximum tolerated dose [81].
NCT00761696	Phase 1	Solid tumor	Saridegib		The maximum tolerated dose (MTD) of saridegib was 160 mg QD within 28-day cycles [82].
NCT01787331	Phase 2	Biochemically relapsed prostate cancer	Itraconazole		One patient (5%) had a > 50% PSA decline [83].
NCT01108094	Phase 2	BCC	Itraconazole		Itraconazole reduced cell proliferation by 45%, HH pathway activity by 65% and reduced tumor area by 24% [84].
NCT00769600	Phase 2	Recurrent non-small cell lung cancer	Itraconazole + pemetrexed	Pemetrexed	Median PFS was 5.5 months (itraconazole) versus 2.8 months (control). There were no evident differences in toxicity between the study arms [85].
NCT00887458	Phase 2	Metastatic castration-resistant prostate cancer	Itraconazole 200 mg daily and 600 mg daily		The PSA PFS rates at 24 weeks were 11.8% in the low-dose arm and 48.0% in the high-dose arm [86].
NCT01919398	Phase 1	Metastatic solid tumor	Taladegib		No dose-limiting toxicities were observed at doses of 100 mg or 200 mg; 3 of the 9 patients evaluable for DLTs at the 400 mg dose level experienced DLTs [83].
NCT01226485	Phase 1	Advanced cancer	Taladegib		The maximum tolerable dose was 400 mg [87].
NCT01546038	Phase 1/2	Acute myeloid leukemia High-risk myelodysplastic syndromes	A: Glasdegib + low-dose ARA-C B: Glasdegib + decitabine C: Glasdegib + daunorubicin + cytarabine		No dose-limiting toxicities (DLT) were observed in arms A and B; 1 DLT (grade 4 neuropathy) occurred in arm C. 46.4% of patient achieved investigator-reported. Among patients ≥55 years old (n = 60), 40.0% achieved complete remission [88,89].
NCT01286467	Phase 1	Solid tumors	Glasdegib		The first-cycle DLT rate at the 640 mg dose level was 33.3%, and the O maximum tolerable dose was estimated to be 320 mg once daily [90].

EP: end point; BCC: basal cell carcinoma; AE: adverse events; DLBCL: diffuse large B-cell lymphoma; iNHL: indolent lymphoma; PCNSL: primary central nervous system lymphoma; CLL: chronic lymphocytic leukemia; laBCC: locally advanced basal cell carcinoma; mBCC: metastatic basal cell carcinoma.

**Table 2:** HhP inhibitors that are being evaluated in clinical trials as of October 2018. Data from www.clinicaltrials.gov.

Study	Phase	Type of Cancer	Experimental Arm	Control Arm	Status
NCT02138929	Phase 1	Advanced gastroesophageal cancer	Sonidegib + everolimus		Active, not recruiting
NCT01485744	Phase 1	Advanced pancreatic adenocarcinoma	Sonidegib + FOLFIRINOX		Active, not recruiting
NCT01431794	Phase 1/2	Pancreatic	Neoadjuvant gemcitabine, nab-paclitaxel and sonidegib	Neoadjuvant gemcitabine, nab-paclitaxel	Active, not recruiting
NCT02151864	Phase 1	Hepatocellular carcinoma	Sonidegib in patients intolerant to sorafenib		Active, not recruiting
NCT02303041	Phase 2	Metastatic basal cell cancer	Sonidegib + buparlisib		Completed
NCT01576666	Phase 1	Advanced solid tumors	Sonidegib + buparlisib		Completed
NCT03434262	Phase 1	Recurrent brain tumors	Sonidegib among others		Recruiting
NCT01787331	Phase 2	Biochemically relapsed prostate cancer	Itraconazole		Active, not recruiting
NCT02735356	Early phase 1	Basal cell cancer	Topical itraconazole		Active, not recruiting
NCT02357836	Early phase 1	Non-small cell lung cancer	Neoadjuvant itraconazole		Recruiting
NCT02749513	Early phase 1	Esophagus	Itraconazole		Recruiting
NCT01835626	Phase 2	Basal cell cancer	Vismodegib + radiotherapy		Recruiting
NCT03052478	Phase 2	Advanced gastric cancer	Vismodegib		Recruiting
NCT01878617	Phase 2	Medulloblastoma	Vismodegib among others		Recruiting
NCT03035188	Phase 2	Basal cell cancer	Neoadjuvant vismodegib		Recruiting
NCT02694224	Phase 2	Triple-negative breast cancer	Neoadjuvant vismodegib + paclitaxel, epirubicin and cyclophosphamide	Neoadjuvant paclitaxel, epirubicin and cyclophosphamide	Recruiting
NCT01791894	Phase 1/2	Basal cell cancer	Arsenic trioxide		Completed
NCT01470248	Phase 2	Advanced small cell lung cancer	Arsenic trioxide		Completed
NCT03503864	Phase 2	Advanced neuroblastoma	Arsenic trioxide + conventional induction chemotherapy	Conventional induction chemotherapy	Recruiting
NCT03466450	Phase 1/2	Glioblastoma	Glasdegib + temozolomide		Recruiting
NCT02530437	Phase 1/2	Localized esophageal or gastroesophageal junction cancer	Taladegib + carboplatin, paclitaxel and radiation		Active, not recruiting
NCT01130142	Phase 1/2	Metastatic pancreatic adenocarcinoma	Saridegib + gemcitabine	Gemcitabine	Completed
NCT01383538	Phase 1	Metastatic pancreatic adenocarcinoma	Saridegib + FOLFIRINOX		Completed
NCT01310816	Phase 2	Metastatic or locally advanced chondrosarcoma	Saridegib	Placebo	Completed

## Abbreviations

The following abbreviations are used in this manuscript:

AKT1	V-akt murine thymoma viral oncogene homolog 1
ATO	Arsenic trioxide
BCC	Basal cell carcinoma
BET	Bromodomain and extra terminal
BRD4	Bromodomain-containing protein 4
CK1	Casein kinase 1
DHH	Desert Hedgehog
EFS	Event-free survival
EGFR	Epidermal growth factor receptor
EMT	Epithelial-to-mesenchymal transition
GLI	Glioma-associated oncogene
GSK3β	Glycogen synthase kinase 3β
HhP	Hedgehog pathway
HIF2α	Hypoxia-inducible factor 2α
HNSCC	Head and neck squamous cell carcinoma
HPI	Hedgehog pathway inhibitors
HR	Hazard ratio
IHH	Indian Hedgehog
IL-1β	Interleukin-1β
KLF4	Kruppel-like factor 4
LSC	Leukemic stem cell
mTOR	Mechanistic target of rapamycin
NF-κB	Nuclear factor kappa light chain enhancer of activated B cells
OCT4	Octamer binding transcription factor 4
ORR	Overall response rate
OS	Overall survival
PFS	Progression-free survival
PKA	Protein kinase A
PTCH1	Patched1
RFS	Relapse-free survival
S6K1	Ribosomal protein S6 kinase 1
SHH	Sonic Hedgehog
SMO	Smoothened
SUFU	Suppressor of fused
TGF-β	Transforming growth factor-beta
TLR	Toll-like receptor
TNF-α	Tumor necrosis factor-α
